# Screening of Young Female Students for Anemia and RhD Blood Group in Vidisha City, India: A Cross-Sectional Study

**DOI:** 10.7759/cureus.100907

**Published:** 2026-01-06

**Authors:** Tina Rai, Richa Nigam, Gayatree Bharti, Deepshikha Verma

**Affiliations:** 1 Department of Pathology, Atal Bihari Vajpayee Government Medical College, Vidisha, IND; 2 Department of Community Medicine, Atal Bihari Vajpayee Government Medical College, Vidisha, IND; 3 Department of Obstetrics and Gynecology, Atal Bihari Vajpayee Government Medical College, Vidisha, IND

**Keywords:** adolescents, anemia, central india, iron deficiency, megaloblastic anemia, rh typing

## Abstract

Background

Young age is a critical period, marked by rapid growth and increased physiological demands. Young girls are particularly vulnerable to anemia due to the onset of menstruation and nutritional deficiencies. Early identification and management are key to preventing long-term health consequences.

Objective

The main objective of this study is to determine the prevalence, severity, and etiological pattern of anemia among young girls, and to assess their Rh blood group status in Vidisha city, India.

Methods

A cross-sectional study was conducted among 200 young girls, aged 17-21 years, at a Government Girls’ College in Vidisha, India. Hemoglobin estimation was done using an automated three-part hematology analyzer. Anemia was classified per the World Health Organization (WHO) criteria. Morphological classification (microcytic hypochromic vs. macrocytic) of anemia, based on hematological indices and peripheral smear findings, was performed, and RhD groups were analyzed.

Results

Anemia was present in 40% of participants. Among anemic girls, 57.5% had mild anemia, 37.5% had moderate anemia, and 5% had severe anemia. Microcytic hypochromic anemia was the predominant type (80%), followed by macrocytic anemia (20%). RhD-positive status was noted in 93% of girls, and RhD-negative status in 7%.

Conclusion

A high burden of anemia exists among young girls in Vidisha city, predominantly microcytic hypochromic. Routine screening and targeted nutritional interventions are essential, and RhD typing awareness is important for future obstetric care.

## Introduction

Young age is a phase of accelerated physical growth and physiological transition, during which nutritional demands increase substantially. Young girls are particularly susceptible to anemia because of the onset of menstruation and heightened iron requirements. Anemia during this stage can lead to reduced physical capacity, impaired cognitive function, decreased academic performance, and increased risk during future pregnancies [1].

In India, most national health initiatives are directed toward children, pregnant women, and older adults, whereas adolescents and young people often receive comparatively less-focused attention. This gap is concerning, as adolescence represents a crucial period for the prevention of nutritional deficiencies that may persist into adulthood. Previous studies have reported a higher prevalence of nutritional anemia among adolescent and young girls, especially at the onset of menarche, which is associated with increased iron loss and unmet dietary requirements [2,3].

Anemia continues to be a major public health and nutritional problem worldwide, disproportionately affecting young females and contributing significantly to morbidity. If not identified and managed at an early stage, anemia can adversely affect the entire life course of women, including an increased risk of maternal complications, adverse pregnancy outcomes, and long-term health impairments [1,4,5].

Nutritional deficiencies during infancy, childhood, and adolescence have far-reaching consequences, with a higher burden observed in low- and middle-income countries. In India, anemia predominantly affects women of reproductive age, lactating mothers, children, and adolescent girls, making it a priority public health concern [4].

In addition to anemia screening, awareness of blood group and RhD status is essential for future obstetric management and transfusion preparedness. However, routine RhD typing is not uniformly emphasized or accessible in adolescent- and young-age health programs in many parts of Central India.

The present study was undertaken to screen young girls in Vidisha city, India, for anemia; assess its prevalence and severity according to the World Health Organization (WHO) criteria; identify the predominant morphological patterns; and determine RhD blood group distribution. The findings aim to provide evidence to support targeted nutritional interventions, early treatment strategies, and increased awareness regarding RhD status among young girls.

## Materials and methods

Study period

The study was conducted over a period of two months, from August 2025 to September 2025, during a screening organized at Government Girls’ Colleges, Vidisha, India. The study colleges were selected through random selection. The universal sampling method was adopted, in which all eligible young girls who gave their consent were included in the study.

Study design and setting

A cross-sectional study was conducted through a camp organized at Government Girls’ College, Vidisha, India, by the Department of Pathology and the Department of Obstetrics and Gynecology of Atal Bihari Vajpayee Government Medical College (ABVGMC), Vidisha, a prominent tertiary care center of central India. The study took place over a period of two months (August-September 2025) and screened a total of 200 female students. The Institutional Ethics Committee of ABVGMC issued approval for this study (approval no. 10/B/6/25/IEC/ABVGMC/Vidisha/2025).

Participants

All volunteer female students of Government Girls’ College (17-21 years) who provided informed consent were included in the present study, while females with a history of recent blood transfusion, acute severe illness, previously diagnosed hemoglobinopathies (including thalassemia), recent gastrointestinal bleeding, or those currently on oral hematinics were excluded to minimize confounding. The study was conducted after institutional ethical approval from ABVGMC, Vidisha.

Questionnaire

A self-designed, semi-structured questionnaire was developed by the investigators specifically for this study to collect socio-demographic details, socioeconomic status, menstrual history, dietary habits, and awareness regarding anemia. The questionnaire was reviewed by subject experts for content validity and is provided as Appendix 1.

The materials required for blood collection, such as disposable syringes and needles, alcohol, gauze or cotton, and collection bottles, were assembled. The subject was instructed to sit alongside the table, keeping their arm on the table with the palm upwards. The median cubital vein was mostly preferred to withdraw 4 mL of blood into an ethylenediaminetetraacetic acid (EDTA) vial. Blood collection was carried out by a qualified team, maintaining good hygiene and safety practices, and was then processed in a Mindray 3-part CBC analyzer (Mindray Medical International, Shenzhen, China). Hemoglobin levels were determined, and the anemic status of adolescent girls was assessed using the WHO classification. An individual adolescent girl was considered anemic if the Hb value was below 12.0 g/dL. Girls with anemia were further categorized into different grades: mild (10-11.9 g/dL), moderate (7-9.9 g/dL), and severe (<7.0 g/dL) [6]. RhD typing was carried out by the manual tube agglutination method using standard anti-D reagent; weak D and partial D variants were not evaluated.

The etiological classification of anemia in this study was based on hematological parameters and peripheral smear findings, and represents a presumptive categorization for screening purposes. Confirmatory biochemical and hemoglobinopathy investigations were beyond the scope of the present study.

Sample size calculation

The sample size was calculated using the standard formula for prevalence studies: $n = \frac{Z^2 \times p \times q}{d^2}$, where n = required sample size, Z = standard normal variate (1.96 at 95% confidence level), p = expected prevalence of anemia among adolescent girls (assumed to be 40% based on previous studies), q = 1 - p, and d = allowable error (7%).

Based on the above calculation, the minimum sample size required was approximately 188, which was rounded to 200 participants for adequate representation and feasibility.

Statistical analysis

The data were compiled and entered into a spreadsheet (Microsoft Excel; Microsoft® Corp., Redmond, WA, USA), and then exported to the data editor of IBM SPSS Statistics for Windows, Version 20 (Released 2011; IBM Corp., Armonk, NY, USA). Quantitative variables were summarized as means and standard deviations.

## Results

A total of 200 young girls, aged 17-21 years, were screened in the present study. The mean age of participants was 18.9 ± 1.2 years, as shown in Table 1.

**Table 1:** Demographic Characteristics of Participants (n = 200)

Variable	Value
Age range (years)	17-21
Mean age (years)	18.9 ± 1.2
Total participants	200

Prevalence of anemia

Out of 200 girls, 80 (40%) were found to be anemic, as per WHO criteria (Hb < 12 g/dL). The remaining 120 (60%) had normal hemoglobin levels.

Severity of anemia

Among the 80 anemic girls, mild anemia (10-11.9 g/dL) was found in 46 (57.5%), moderate anemia (7-9.9 g/dL) in 30 (37.5%), and severe anemia (<7 g/dL) in 4 (5%). Mild anemia constituted the largest proportion, while severe anemia was the least common, as shown in Table 2.

**Table 2:** Severity of Anemia in Present Study WHO, World Health Organization

Severity (WHO Criteria)	Frequency (n)	Percentage (%)
Mild (10-11.9 g/dL)	46	57.5%
Moderate (7-9.9 g/dL)	30	37.5%
Severe (<7 g/dL)	4	5%
Total	80	100%

Morphological classification of anemia

Among the anemic girls (n = 80), 64 (80%) were suffering from microcytic hypochromic anemia, while 16 (20%) had macrocytic anemia. Thus, microcytic hypochromic anemia - the most common type, usually caused by iron deficiency - was the predominant cause in this adolescent population, as shown in Table 3.

**Table 3:** Morphological Classification of Anemia

Type of Anemia	Frequency (n)	Percentage (%)
Microcytic hypochromic anemia	64	80%
Macrocytic anemia	16	20%
Total	80	100%

Hematological indices among iron-deficient anemic females are shown in Table 4.

**Table 4:** Hematological Indices Among Anemic Young Girls (n = 80) MCV, Mean Corpuscular Volume; MCH, Mean Corpuscular Hemoglobin; MCHC, Mean Corpuscular Hemoglobin Concentration; RDW, Red Cell Distribution Width; IDA, Iron Deficiency Anemia

Parameter	Mean ± SD	Reference Range	Interpretation
MCV (fL)	74.2 ± 8.4	80-100 fL	Low MCV supports microcytic anemia
MCH (pg)	23.8 ± 3.1	27-32 pg	Low MCH suggests hypochromia
MCHC (g/dL)	29.4 ± 1.8	32-36 g/dL	Low MCHC indicates hypochromic RBCs
RDW (%)	16.9 ± 2.4	11.5-14.5%	Elevated RDW indicates anisocytosis typical in IDA

Rh typing

All 200 participants were tested for Rh blood group status. Of the study participants, 186 girls (93%) were Rh-positive, while 14 (7%) were Rh-negative. The distribution is consistent with general Indian population patterns, where the frequency of Rh-negative individuals remains low.

Participants from lower socioeconomic strata showed a higher proportion of anemia compared to those from higher socioeconomic groups. These findings represent descriptive observations from the study population.

## Discussion

The present study demonstrates a substantial prevalence of anemia (40%) among young girls in Vidisha city, Central India, reaffirming anemia as a significant public health concern in this age group. The majority of cases were categorized as mild or moderate anemia, suggesting a chronic nutritional deficiency rather than an acute illness. Similar prevalence patterns have been reported in studies conducted in different regions of India, indicating that adolescent anemia remains a widespread issue across diverse geographic settings [1,2].

Microcytic anemia can result from multiple etiologies, including iron deficiency, thalassemia syndromes, anemia of chronic disease/inflammation, sideroblastic anemia, other nutritional deficiencies (such as copper deficiency or excess zinc), and exposure to certain medications or alcohol. Although iron deficiency remains the most common cause globally, particularly among adolescent girls, other causes cannot be excluded based solely on red cell indices and peripheral smear findings. Therefore, the microcytic hypochromic anemia observed in the present study represents a morphological pattern rather than a definitive etiological diagnosis, and further biochemical and specialized investigations are required for accurate cause determination [4]. Also, red cell distribution width (RDW) in a higher range suggests dynamic pathology and, hence, mainly indicates iron deficiency anemia; however, other hemoglobinopathies, having static pathology, show RDW in an almost normal range.

Microcytic hypochromic anemia was identified as the predominant morphological type, accounting for 80% of anemic cases in the present study. This finding is consistent with existing literature, which identifies iron deficiency as the most common cause of anemia among adolescents in low- and middle-income countries. Given that iron deficiency anemia has been ranked among the leading modifiable risk factors contributing to the global disease burden, early detection and intervention remain critical [7]. Although iron deficiency is reported as the most common cause of microcytic anemia in adolescents globally, the present study reports only morphological patterns, and etiological causes could not be confirmed.

Adolescence is characterized by rapid physical growth and the establishment of regular menstrual cycles, both of which substantially increase iron requirements. Inadequate dietary intake, poor absorption of iron, limited consumption of iron-rich foods, and socioeconomic constraints further contribute to the development of anemia during this period. Persistent iron deficiency during adolescence has been shown to negatively affect cognitive performance, physical endurance, and academic achievement, and may increase the risk of adverse maternal and fetal outcomes in later life [8,9].

The present study observed a higher prevalence of anemia among participants belonging to lower socioeconomic strata, supporting previous evidence that socioeconomic status, parental education, dietary practices, household hygiene, and awareness regarding nutrition play an important role in determining anemia prevalence. Girls from higher socioeconomic backgrounds tended to have normal hemoglobin levels, or milder forms of anemia, likely due to better access to nutritious diets and health information [10,11].

Menstrual blood loss is often considered a major contributor to anemia in adolescent girls; however, no significant association was observed between anemia prevalence and the duration of menstrual flow in the present study. Similar findings have been reported in earlier studies, suggesting that factors other than menstrual duration - such as dietary intake and overall nutritional status - may play a more influential role [12,13].

Megaloblastic anemia constituted 20% of anemic cases in this study, indicating deficiencies of folate and vitamin B12. This highlights inadequate dietary diversity and underscores the importance of addressing micronutrient deficiencies, in addition to iron supplementation. School- and college-based nutrition education programs may play a vital role in improving dietary awareness and preventing such deficiencies [14,15].

The Rh-negative prevalence of 7% observed in the present study is consistent with reported Indian population data. Early identification of Rh status during adolescence is beneficial for future reproductive counseling and can help prevent Rh incompatibility-related complications, including hemolytic disease of the newborn, through timely antenatal interventions.

The strengths of this study include its community-based screening approach and the combined evaluation of anemia severity, etiological patterns, and Rh typing. Overall, the findings emphasize the need for routine anemia screening, nutritional counseling, and targeted supplementation programs for adolescent girls. Integrating such initiatives into educational institutions may significantly contribute to improving adolescent health and reducing future maternal morbidity.

Limitations

The present study has certain limitations. The sample size was limited to students from a single educational institution, which may affect the generalizability of the findings. Biochemical parameters, such as serum ferritin, vitamin B12, and folate levels, could not be assessed due to the camp-based nature of the screening, and anemia etiology was inferred using hematological indices. However, after screening, these girls were referred to a tertiary center for proper investigations and management.

Hemoglobin electrophoresis/high-performance liquid chromatography (HPLC) was not performed; therefore, the thalassemia trait could not be conclusively excluded. Additionally, dietary intake was assessed through self-reported information, which may be subject to recall bias. The association between socioeconomic status and hemoglobin levels was based on descriptive analysis. The questionnaire-based data were self-reported and lacked detailed dietary quantification, which may have introduced recall bias. As menstrual blood loss was not quantitatively assessed, any association between menstruation and anemia in this study should be interpreted cautiously. 

## Conclusions

A substantial proportion of young girls continue to suffer from anemia, with microcytic hypochromic anemia being the predominant morphological pattern. The findings underscore the need for routine screening, nutritional counseling, and further etiological evaluation, where feasible. This highlights the ongoing burden of nutritional anemia in this vulnerable age group and underscores the need for early identification through routine screening programs. Implementation of iron-folic acid supplementation, along with dietary counseling and structured awareness initiatives at the school and college level, is essential to prevent long-term health consequences. While universal iron-folic acid supplementation remains a practical public health approach in high-burden settings, screening-based targeted supplementation is preferable where diagnostic facilities are available. In addition, incorporation of Rh typing during adolescence provides valuable information for future reproductive counseling and maternal health planning, thereby contributing to improved obstetric outcomes.

## Appendices

Appendix 1: Semi-structured questionnaire used in the study

This questionnaire was self-developed by the investigators for the present study and was not adapted from any previously published tool.

Section A: Socio-Demographic Details

Age (in completed years): ___

Educational level: ☐ Undergraduate ☐ Postgraduate

Place of residence: ☐ Urban ☐ Rural

Socioeconomic status (as per the modified Kuppuswamy scale): ☐ Lower ☐ Middle ☐ Upper

Section B: Menstrual History

Age at menarche (years): ___

Duration of menstrual cycle: ☐ <3 days ☐ 3-5 days ☐ >5 days

History of excessive menstrual bleeding: ☐ Yes ☐ No

Section C: Dietary History

Type of diet: ☐ Vegetarian ☐ Mixed

Frequency of consumption of iron-rich foods (green leafy vegetables, pulses, jaggery): ☐ Daily ☐ Weekly ☐ Occasionally

History of iron or folic acid supplementation: ☐ Yes ☐ No

Section D: Awareness About Anemia

Awareness about anemia: ☐ Yes ☐ No

Knowledge regarding symptoms of anemia: ☐ Yes ☐ No

## References

[1] Chandrakumari AS, Sinha P, Singaravelu S, Jaikumar S (2019). Prevalence of anemia among adolescent girls in a rural area of Tamil Nadu, India. J Family Med Prim Care.

[2] Vijayshree M, Shobha B, Nitin M (2023). Assessment of anemia and malnutrition among adolescent in Kalyan Karnataka region of Karnataka. Clin Epidemiol Glob Hlth.

[3] Kaur S, Deshmukh PR, Garg BS (2006). Epidemiological correlates of nutritional anemia in adolescent girls of rural Wardha. Indian J Community Med.

[4] Anand T, Rahi M, Sharma P, Ingle GK (2014). Issues in prevention of iron deficiency anemia in India. Nutrition.

[5] (2025). Adolescent and young adult health. https://www.who.int/news-room/fact-sheets/detail/adolescents-health-risks-and-solutions.

[6] Ahmed A, Mohammed A (2022). Anemia and its associated factor among adolescent schoolgirls in Godey and Degehabur Council Somali Region, eastern Ethiopia. BMC Nutr.

[7] Mantadakis E, Chatzimichael E, Zikidou P (2020). Iron deficiency anemia in children residing in high and low-income countries: risk factors, prevention, diagnosis and therapy. Mediterr J Hematol Infect Dis.

[8] More S, Shivkumar VB, Gangane N, Shende S (2013). Effects of iron deficiency on cognitive function in school going adolescent females in rural area of central India. Anemia.

[9] Gupta A, Parashar A, Thakur A, Sharma D (2012). Anemia among adolescent girls in Shimla Hills of north India: does BMI and onset of menarche have a role?. Indian J Med Sci.

[10] Kim JY, Shin S, Han K (2014). Relationship between socioeconomic status and anemia prevalence in adolescent girls based on the fourth and fifth Korea National Health and Nutrition Examination Surveys. Eur J Clin Nutr.

[11] Habib N, Abbasi SS, Aziz W (2020). Analysis of societal determinant of anemia among adolescent girls in Azad Jammu and Kashmir, Pakistan. Anemia.

[12] Saratha A, Singh Z, Datta SS (2010). Prevalence of anaemia among young adult female students in a medical teaching institution in Pondicherry. Indian J Matern Child Health.

[13] Sonawane SV, Todkar SS, Mulaje S (2025). Prevalence of anemia and factors affecting anemia among adolescent girls under field practice area of rural health training centre of western Maharashtra. Int J Community Med Public Health.

[14] Verma SK, Khanum RS (2021). A study on prevalence of anemia among late adolescent girls in JSS schools and colleges of rural and urban Mysuru district, Karnataka, India. Int J Comm Med PublHlth.

[15] Gillespie B, Katageri G, Salam S (2023). Attention for and awareness of anemia in adolescents in Karnataka, India: a qualitative study. PLoS One.

